# Clinical profile and long-term predictors of mortality in idiopathic acute pulmonary thromboembolism

**DOI:** 10.21542/gcsp.2024.57

**Published:** 2024-12-31

**Authors:** Rujuta Parikh, Iva Patel, Vishal Patel, Pooja Vyas, Hasit Joshi, Utsav Patel, Sagar Ghetiya

**Affiliations:** U. N. Mehta Institute of Cardiology and Research Centre (UNMICRC), Civil Hospital Campus Asarwa, Ahmedabad-380016, Gujarat India

## Abstract

**Background:** Unprovoked venous thromboembolism is a poorly understood entity. Clinical risk factors and future outcomes are not well recognized in this subgroup of patients. Various pathogenic mechanisms like inflammation and athero-thrombosis have been put forth but remain investigative. Our study aimed to determine the clinical profile and predictors of mortality in patients with idiopathic pulmonary embolism.

**Methods:** Our single centre observational study included 510 consecutive patients with symptomatic unprovoked venous thromboembolism. Pulmonary embolism (PE) patients were initially categorized based on the presence or absence of deep vein thrombosis (DVT). Subsequently, the patients were further sub-grouped according to mortality, and the association between clinical parameters and death was evaluated through regression analysis.

**Results:** The in-hospital mortality of patients with unprovoked pulmonary embolism was 15.9% and 25.76% at three year follow up. Significantly higher number of patients with diabetes, hypertension, dyslipidaemia, lower TAPSE (tricuspid *annular plane systolic excursion)* and PASP (pulmonary arterial systolic pressure) were found in mortality patients compared to survivor patients. On regression analysis we found significant association of higher odds of age OR = 1.1 (1.05–1.23), diabetes OR = 2.47 (1.28–4.79), hypertension OR = 2.25 (1.19–4.26) and lower odds of thrombolysis OR = 0.38 (0.11–0.59) with mortality. On Kaplan Meier survival analysis, the log value of <0.05 showed significantly higher mortality in patients who were not thrombolyzed.

**Conclusion:** Various short and long-term predictors of mortality exist for pulmonary embolism. Cardiovascular risk factors play a mediating role in venous thromboembolism and also serve as predictors for long-term mortality. Therefore, modifying these risk factors can potentially result in a reduction in long-term mortality.

## Introduction

Incidence of pulmonary thromboembolism has been reported in one study as being 0.52–0.69 per 1000 people per year^1^. Almost one third of these patients do not have any identifiable provocative risk factor (malignancy, immobilization, pregnancy, heart failure, trauma or stroke) and are considered as idiopathic pulmonary embolism^2^. Mortality due to pulmonary embolism is estimated to occur in 11.4% of patients at two weeks and 17.4% at 3 months^3^. However, the mortality of pulmonary embolism with cardiogenic shock can be as high as 25 to 30%^4^. Little is known about the risk factors and mortality predictors of idiopathic pulmonary embolism. Our study aimed to determine the clinical profile and predictors of mortality in this subset of patients.

## Methods

**Figure 1.**
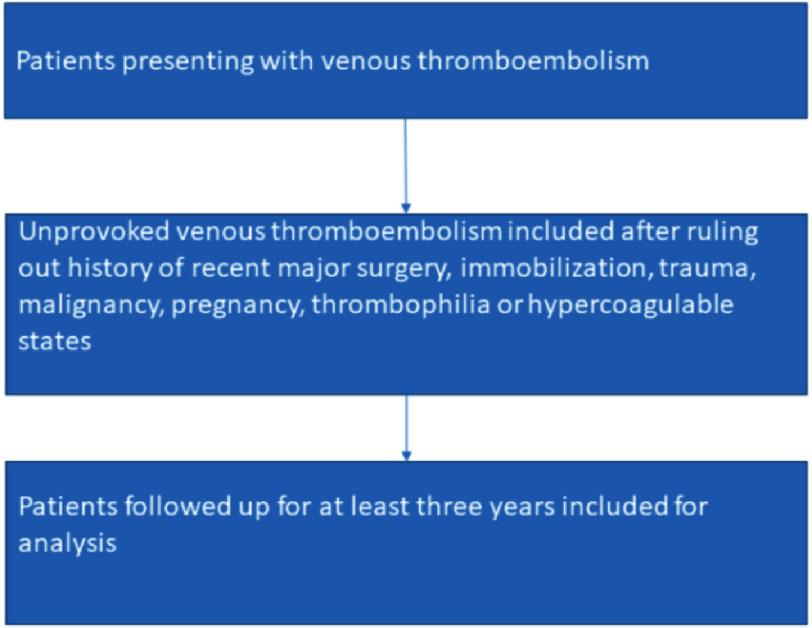
Flow chart of the study population.

This observational study enrolled 510 consecutive patients with symptomatic unprovoked venous thromboembolism in the form of deep vein thrombosis and/or acute pulmonary embolism, presenting to our institute between 2018 to 2020, and who were followed up for a minimum period of 3 years (Figure 1).

 The information collected included demographic data, symptoms on presentation, risk factors for venous thromboembolism, therapeutic management offered to them and clinical follow up at 3 years. The study was approved by institutional ethics committee and written informed consent was obtained from all participants in accordance to the institute’s ethics committee.

Pulmonary embolism was proven by CT pulmonary angiography in all patients. They were classified into Massive, Submassive and Low risk based on hemodynamic and blood investigations. Massive pulmonary embolism was defined as evidence of pulmonary embolism in either main pulmonary artery (MPA) or one of its main branches (RPA or LPA) on CTPA with arterial hypotension (systolic blood pressure less than 90 mm Hg) or signs of poor end organ perfusion. Submassive pulmonary embolism was defined as thrombosis in segmental branches of RPA or LPA on CTPA with hypoxia, RV dysfunction or elevated cardiac markers (Troponin I and BNP) without hypotension or compromised end organ perfusion. Others were classified as low risk.

Unprovoked venous thromboembolism was defined as absence of immobilization, trauma, pregnancy, thrombophilia or intake of prothrombotic drugs like oral contraceptive pills, hormone replacement therapy, androgens etc. Active malignancy or history of having taken treatment for same in last one year was ruled out.

### Statistical analysis

A comparison of parametric values between two groups was performed using the independent sample *t*-test. Categorical variables were compared using the https://www.sciencedirect.com/topics/medicine-and-dentistry/chi-square-test. Survival plot analysis in both groups were performed using Kaplan Meier method and comparisons between groups were performed using Log Rank test (Mantel-Cox). The forest plot was evaluated to assess the association of clinical variables and fatal pulmonary embolism. A probability value (*p*-value) less than 0.05 was considered statistically significant. All statistical analysis was carried out using the SPSS (Statistical Package for the Social Science) program *vs* 20.

## Results

### Baseline characteristics

510 patients presenting with symptomatic unprovoked venous thromboembolism were studied. Their demographic variables are detailed in Table 1. Mean age of our patients was 49.42 ± 17.45 years. 59.6% were males and 40.4% were females. History of prior surgery more than three months prior to presentation was present in 19.2% and history of remote malignancy without any active disease was present in 1.4%. 47.8% of patients presented with proximal DVT. While 37.7% of patient having proximal DVT developed pulmonary embolism, none of the patients with distal DVT alone developed it.

**Table 1:** Clinical profile of patients presenting with venous thromboembolism.

Variable	Venous thromboembolism (*N* = 510)	Pulmonary embolism (*N* = 295)	*P*-value
Age	49.42 ± 17.45	50.39 ± 17.30	0.4461
Female	204 (40.4%)	113 (38.31%)	0.6896
Male	304 (59.6%)	182 (61.69%)	
Diabetes	68 (13.33%)	45 (15.25%)	0.5152
Hypertension	99 (19.41%)	50 (16.95%)	0.4397
Smoker	117 (22.94%)	77 (26.10%)	0.3551
Dyslipidaemia	92 (18.04%)	70 (23.73%)	0.0623
History of surgery	109 (19.2%)	58 (19.66%)	0.6264
Malignancy	08 (1.4%)	01 (0.33%)	0.2109
Proximal DVT	271 (47.8%)	97 (32.88%)	<0.0001
Distal DVT	03 (0.5%)	00	0.4718
Both proximal and distal DVT	23 (4.1%)	14 (4.74%)	0.9839
Massive PE	71 (13.92%)	71 (24.07%)	0.0004
Sub-massive PE	65 (12.74%)	65 (22.03%)	0.0900
Low-risk PE	159 (28.2%)	159 (53.90%)	<0.0001
BNP	436.46 ± 715.25	461.13 ± 650.73	0.6263
Pro BNP	5336.88 ± 7607.11	4250.80 ± 5746.33	0.0321
Creatinine	1.47 ± 1.20	1.18 ± 0.61	0.0001
CRP	55.60 ± 71.08	56.83 ± 73.41	0.8153
D-dimer	4168.80 ± 3113.60	4331.93 ± 3126.75	0.4747
Troponin I	674.22 ± 4911.42	410.72 ± 3318.40	0.4127
eGFR	75.93 ± 42.82	74.88 ± 41.52	0.7347
Procalcitonin	4.41 ± 12.57	4.31 ± 13.33	0.9157
IVC filter	24 (4.4%)	12 (4.07%)	0.8064
Thrombolysis	56 (10.98%)	45 (15.25%)	0.0911

**Notes.**

IVC inferior vena cava GFR Glomerular filtration rate CRP C-reactive protein BNP B-type natriuretic peptide DVT Deep vein thrombosis PE Pulmonary embolism

Table 2 presents the comparison of blood investigations between pulmonary embolism patients with DVT and without DVT. Hypertension and dyslipidaemia, was found significantly higher in PE patients with DVT. CRP was significantly more in patients with DVT and PE suggesting inflammation causing extensive thrombosis (84.79 *vs* 41.35; *p* = 0.001). Other blood investigation in the table were found evenly distributed between both groups.

**Table 2:** Clinical profile of patients with acute pulmonary embolism with and without DVT.

Variables	PE without DVT (*N* = 182)	PE with DVT (*N* = 113)	*P* value
Age	48.95 ± 17.69	52.70 ± 16.48	0.0613
Male	115 (63.2%)	67 (59.3%)	0.2925
Female	67 (36.8%)	46 (40.7%)	
Diabetes	24 (13.2%)	21 (18.6%)	0.1424
Hypertension	24 (13.2%)	26 (23%)	0.0212
Smoker	48 (26.4%)	29 (25.7%)	0.5025
Dyslipidemia	37 (20.3%)	33 (29.2%)	0.05
BNP	408.47 ± 611.93	559.96 ± 715.55	0.2324
Pro BNP	5171 ± 6658.61	2717 ± 4610.08	0.5956
Creatinine	1.22 ± 0.51	1.3 ± 0.78	0.2897
CRP	41.35 ± 59.89	84.79 ± 86.8	0.0010
D-dimer	4061.22 ± 3086.8	4756.49 ± 3159.33	0.1030
Troponin I	588.78 ± 4198.62	119.14 ± 339.46	0.2881
eGFR	77.26 ± 46.47	71.09 ± 31.94	0.2352
Procalcitonin	5.27 ± 15.17	2 ± 8.33	0.3385

### Fatal pulmonary embolism

At 3 years of follow up, 76 (25.76%) patients of pulmonary embolism had died. 15.9% were in hospital mortality. The patients who died were older and had comorbidities in the form of diabetes, hypertension and dyslipidaemia. Patients who were thrombolysed had an immediate lower risk of mortality as compared to those who did not receive thrombolysis (Table 3).

**Table 3:** Comparison of clinical profile between live and dead patients.

Variables	Live (*N* = 219)	Dead (*N* = 76)	*P* value
Age	49.05 ± 16.61	54.22 ± 18.74	0.02
Female	78 (35.6%)	35 (46.1%)	0.0712
Male	141 (64.4%)	41 (53.9%)	
Diabetes	26 (11.9%)	19 (25%)	0.01
Hypertension	30 (13.7%)	20 (26.3%)	0.01
Smoker	54 (24.7%)	23 (30.3%)	0.2091
dyslipidaemia	46 (21%)	24 (31.6%)	0.05
History of Surgery	38 (17.4%)	20 (26.3%)	0.0612
IVC filter	09 (4.1%)	03 (3.9%)	0.6342
Malignancy	00	01 (1.3%)	0.2582
Heart Rate	107.01 ± 9.9	123.15 ± 10.09	<0.0001
Blood Pressure	112.23 ± 10.21	94.71 ± 10.15	<0.0001
SpO2 on presentation	93.67 ± 5.1	88.21 ± 6.7	<0.0001
TAPSE	18.51 ± 2.31	14.16 ± 3.1	<0.0001
PASP	30.05 ± 5.7	42.82 ± 5.3	<0.0001
Massive	52 (23.7%)	19 (25%)	0.8649
Sub-massive	47 (21.5%)	18 (23.7%)	
Low-risk	120 (54.8%)	39 (51.3%)	
Thrombolysis	182 (83.1%)	08 (10.5%)	<0.0001

**Notes.**

Spo2 Saturation of Peripheral Oxygen TAPSE Tricuspid Annular Plane Systolic Excursion PASP pulmonary arterial systolic pressure

**Figure 2.**
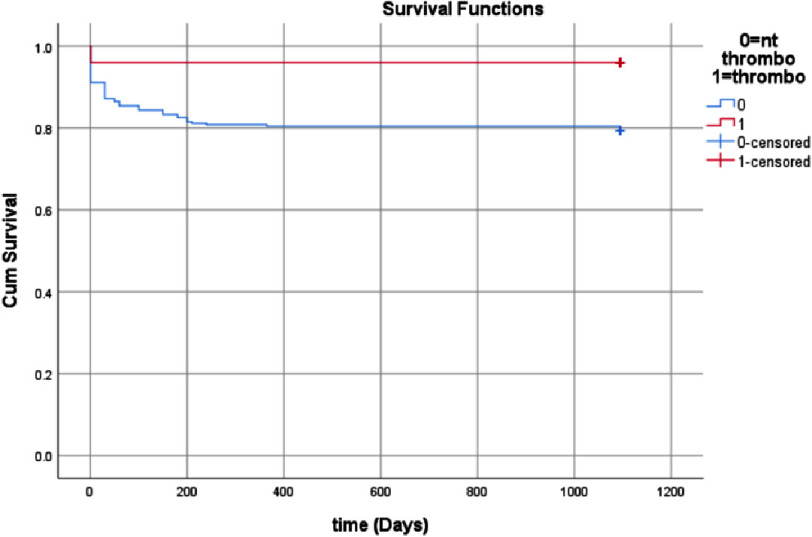
Survival curve for thrombolyzed and non-thrombolyzed patients.

Figure 2 shows the Kaplan Meire curve for patients who were thrombolized and patients who were not thrombolyzed. The log rank value of chi square shows 3.77 with *P* value 0.05.

Table 3 presents the comparison of clinical profile between live and death patients. Cardiac risk factors like higher age, diabetes, hypertension, and dyslipidaemia were found significantly higher in patients with mortality. Diabetes is the link between oxidative stress, hypercoagulability and vascular thrombosis. Studies have put forth various mechanisms including upregulated tissue factor expression by endothelium in diabetics, elevated levels of factor VII and thrombin, elevated interleukin-6 that increases fibrinogen production from liver and a more compact clot resistant to fibrinolysis^5–7^. Systemic hypertension has also shown to modify all three components of Virchow’s triad implicated in venous thromboembolism. It may seem counterintuitive that pulsatile raised blood pressure should cause greater thrombotic complications as compared to haemorrhagic complications and this is often called the “Birmingham Paradox”. However, hypertension causes endothelial dysfunction, an increase in thickness of tunica media, platelet activation and changes in blood rheology causing a prothrombotic state^8,9^. Systolic blood pressure, SPO_2_ on presentation, TAPSE, and PASP were found significantly lower in patients with mortality. The survival group exhibited a higher incidence of patients who underwent thrombolysis.

### Clinical variables association with mortality

**Figure 3.**
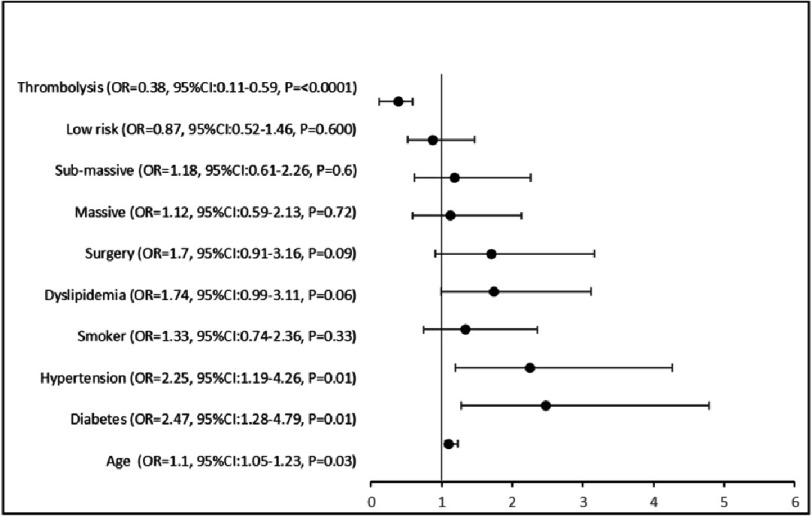
Association of clinical variables and mortality.

Higher odds of older age, hypertension and diabetes were found to be clinical predictors of mortality while the significantly lower odds of thrombolysis were seen for mortality on regression analysis (Figure 3).

## Discussion

The prevalence of symptomatic pulmonary embolism in patients with proximal DVT is estimated to be 40 to 50%^10^. Even though risk factors of venous thromboembolism have been studied, it is as yet unclear as to the difference in initial clinical presentation; with some patients presenting with only DVT or pulmonary embolism, some presenting with both and still others with DVT developing pulmonary thromboembolism despite being on anticoagulation. Survival is significantly lower in patients who develop pulmonary embolism. Pulmonary embolism is an independent predictor of reduced survival at three months. Older age, higher BMI, malignancy, congestive cardiac failure, neurologic disease, chronic lung disease and recent surgery were also found to predict poor short and long term survival in patients with VTE^11^.

The role of inflammation in the pathogenesis of VTE is still investigative. Risk factors such as malignancy, surgery, autoimmune disease and sepsis are thought to modulate thrombosis by inflammation. CRP levels have been associated with increased risk of mortality, RV dysfunction and recurrent venous thromboembolism^12^. Our study suggests that inflammation maybe associated with more extensive venous thromboembolism.

The main finding in our study is that comorbidities like older age, hypertension, diabetes mellitus and dyslipidaemia are predictors of mortality in patients presenting with idiopathic acute pulmonary embolism. However, sPESI, a validated score predictor of mortality, does not include hypertension, diabetes mellitus and dyslipidaemia^13^. It considers chronic heart failure amongst other parameters for predicting mortality at 30 days. However, long term mortality predictors are likely to be different. Studies have proven that long term mortality is associated with various comorbid conditions like malignancy, diabetes mellitus and coronary artery disease^14,15^.

A study by Rodriguez-Nunez et al. examined patients with pulmonary embolism who had no previously known risk factors. The researchers found that advanced age, hypertension, and dyslipidemia were significantly associated with these cases. Moreover, they observed that the risk of pulmonary embolism increased as the number of these factors increased. While there was no difference in short term mortality between patients with pulmonary embolism with and without known identifiable risk factors for similar sPESI scores, long term mortality was higher in elderly and patients with hypertension and dyslipidaemia^16^.

Obesity has consistently been associated with immobilization, inflammation and hypercoagulability. There is increased thrombin formation and decreased fibrinolysis^17^. A large metanalysis showed that cardiovascular risk factors like obesity (OR: 2.33), arterial hypertension (OR: 1.51), diabetes mellitus (OR: 1.42), smoking (OR: 1.18) and hypercholesterolemia (OR: 1.16), significantly increased the risk of venous thromboembolism^18^. Adipose tissue also demonstrate altered signalling as a consequence of overnutrition. They secrete tissue factor and plasminogen activator inhibitor that promotes a prothrombotic state^19^.

Patients who have both diabetes and pulmonary embolism tend to experience more severe clinical symptoms and outcomes. They tend to have more comorbidities (such as coronary artery disease), are more likely to have undergone prior operative procedures, and are more likely to present with tachycardia, right ventricular dysfunction and hemodynamic instability. Patients presenting with higher glycemia levels (>152 mg/dL on admission), had worse clinical outcomes^20^. Consequently, diabetic patients experience longer hospital stays and higher in-hospital mortality than non-diabetic patients^21^.

Diabetes mellitus is a long standing inflammatory state with primarily endothelial dysfunction increasing the risk of both atherothrombosis and venous thrombosis. Elevated levels of C-reactive protein, TNF-alpha, IL-6 and vWF have been demonstrated in diabetic population as compared to non-diabetics^22^. Increased expression and transcription of coagulation factors and increased susceptibility to form fibrin network has been noted. The fibrin mesh is denser due to post-translational glycation and oxidation^23^. Also, it has smaller pore size and has higher branching points making it resistant to lysis. In addition, levels of plasminogen activator inhibitor-1 is raised in diabetic patients, further impairing fibrinolysis^24^.

The biological plausibility of inflammation and altered coagulation system mediating both arterial and venous thrombosis is well established. It is reasonable to suppose that cardiovascular risk factors that contribute to atherosclerosis also mediate venous thrombosis. Our study adds the possible long-term impact on mortality mediated by these factors and the need to modify them.

## Limitations

This is a single centre study in Western India and hence, results may not be generalized to population. Even though, there is evidence to show inflammation and pro-coagulation milieu to cause venous thromboembolism, randomized trials are required to prove their clinical effect in secondary prevention.

## Conclusion

There are different short and long term mortality predictors of pulmonary embolism. Cardiovascular risk factors mediate venous thromboembolism and also predict long-term mortality. Modification of these risk factors can thus lead to a reduction in long-term mortality.

## Conflicts of Interest

The authors declare no potential conflicts of interest with respect to the research, authorship, and/or publication of this article.

## Funding sources

The author(s) received no financial support for the research, authorship, and/or publication of this article.
